# Selective hydrogenolysis of the Csp^2^–O bond in the furan ring using hydride–proton pairs derived from hydrogen spillover†

**DOI:** 10.1039/d4sc05751a

**Published:** 2024-10-28

**Authors:** Fangfang Peng, Bin Zhang, Runyao Zhao, Shiqiang Liu, Yuxuan Wu, Shaojun Xu, Luke L. Keenan, Huizhen Liu, Qingli Qian, Tianbin Wu, Haijun Yang, Zhimin Liu, Jikun Li, Bingfeng Chen, Xinchen Kang, Buxing Han

**Affiliations:** a Beijing National Laboratory for Molecular Sciences, CAS Laboratory of Colloid and Interface and Thermodynamics, CAS Research/Education Center for Excellence in Molecular Sciences, Center for Carbon Neutral Chemistry, Institute of Chemistry, Chinese Academy of Sciences Beijing 100190 China hanbx@iccas.ac.cn kangxinchen@iccas.ac.cn chenbf@iccas.ac.cn jikunli@iccas.ac.cn; b School of Chemical Sciences, University of Chinese Academy of Sciences Beijing 101408 P. R. China; c Department of Chemical Engineering, School of Engineering, The University of Manchester Oxford Road Manchester M13 9PL UK; d Diamond Light Source, Harwell Science Campus Oxfordshire OX11 0DE UK; e Beijing National Laboratory for Molecular Sciences, Key Laboratory of Photochemistry, Institute of Chemistry, Chinese Academy of Sciences Beijing 100190 China; f Shanghai Key Laboratory of Green Chemistry and Chemical Processes, State Key Laboratory of Petroleum Molecular & Process Engineering, School of Chemistry and Molecular Engineering, East China Normal University Shanghai 200062 China

## Abstract

Selective hydrogenolysis of biomass-derived furanic compounds is a promising approach for synthesizing aliphatic polyols by opening the furan ring. However, there remains a significant need for highly efficient catalysts that selectively target the Csp^2^–O bond in the furan ring, as well as for a deeper understanding of the fundamental atomistic mechanisms behind these reactions. In this study, we present the use of Pt–Fe bimetallic catalysts supported on layered double hydroxides [PtFe_*x*_/LDH] for the hydrogenolysis of furanic compounds into aliphatic alcohols, achieving over 90% selectivity toward diols and triols. Our findings reveal that the synergy between Pt nanoparticles, atomically dispersed Pt sites and the support facilitates the formation of hydride-proton pair at the Pt^*δ*+^⋯O^2−^ Lewis acid–base unit of PtFe_*x*_/LDH through hydrogen spillover. The hydride specifically targets the Csp^2^–O bond in the furan ring, initiating an S_N_2 reaction and ring cleavage. Moreover, the presence of Fe improves the yield of desired alcohols by inhibiting the adsorption of vinyl groups, thereby suppressing the hydrogenation of the furan ring.

## Abstract 2

We propose a strategy for the hydrogenolysis of furan rings using PtFe_*x*_/LDH catalysts. Mechanism studies reveal that the hydride attacks the carbon atom of the Csp^2^–O bond, initiating an S_N_2 reaction that results in ring-opening products formation.

## Graphical abstract

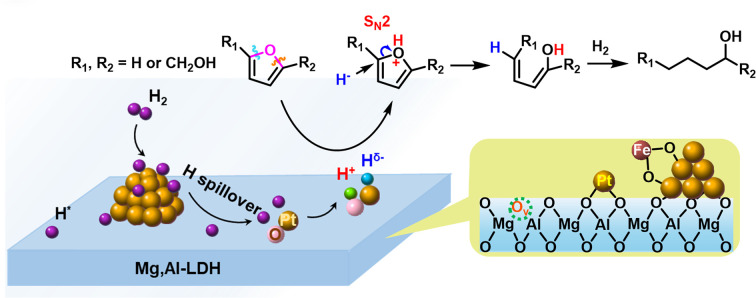

## Introduction

Due to the depletion of fossil resources and the importance of carbon cycling, the conversion of biomass into valuable chemicals has received much attention.^1–3^ Furanic compounds, such as furfural (FFR), furfural alcohol (FA) and 5-hydroxymethylfurfural (HMF), derived from the cellulose hydrolysis, represent crucial platform molecules in biomass conversion. Hydrogenolysis of furanic compounds, achieved through the cleavage of Csp^2^–O bonds in the furan ring, is a highly effective method for producing diols or triols, which are crucial precursors for a range of applications, including polyurethanes, polyesters, polymeric plasticizers and low-toxic microbicides.^4–6^ Supported Pt catalysts have proven effective for the selective hydrogenolysis of furanic compounds, including FFR, FA, HMF, furancarboxylic acids with factors such as composition, metal dispersion, and support all playing significant roles in overall catalytic performance.^7–14^ Despite decades of extensive research aimed at improving the selectivity of diols or triols, achieving a comprehensive understanding of the fundamental atomistic mechanisms that govern the cleavage of Csp^2^–O bonds in the furan ring remains a critical objective.

In addition to the active sites of catalysts, the selectivity for ring-opening products is also associated with the active hydrogen species. In catalytic hydrogenation reactions, the dissociation of H_2_ determines both activity and selectivity. It is widely accepted that on extended metal surfaces (metal–metal sites), H_2_ dissociation tends to favor the homolytic pathway. In contrast, when the local coordination structures of the metal centers involve Lewis acid–base units, H_2_ molecules are more inclined to undergo heterolytic dissociation at these sites, forming hydride–proton pairs.^15–17^ The resulting hydride–proton pairs facilitate the hydrogenation of polar bonds since polar bonds are excellent acceptors for both hydrides and protons.^15,16^ However, the relatively high energy barrier associated with the direct heterolytic pathway may lead to a sluggish hydrogenation kinetics compared with the barrierless homolytic dissociation of H_2_ on metal ensembles.^15^ Hydrogen spillover involves the homolytic dissociation of H_2_ on metals, where the resulting active H* species migrate to either the support or the metal sites. This migration can lead to charge separation into protons and/or hydrides.^18–21^ Specifically, we can infer from the local coordination structures of H_2_ heterolytic dissociation that proton and hydride pairs could form through charge separation of the active H* species when Lewis acid–base units are present on the catalyst.

Over the past decades, layered double oxides (LDOs) and hydroxides (LDHs) have made significant contributions to high-value upgrading reactions of biomass resources.^22^ Due to the availability of acid–base sites, they are excellent candidates for generating protons and hydrides *via* spillover. Herein, we developed Mg,Al-LDH-supported Pt/Fe catalysts (PtFe_*x*_/LDH) for the hydrogenolysis of biomass-derived furanic compounds into their corresponding ring-opening alcohol products, achieving diol/triol yields exceeding 90% with complete conversion of the furanic compounds. The hydrogenolysis process of the Csp^2^–O bond was elucidated through spectroscopic measurements, Kinetic isotope experiment analysis, and density functional theory (DFT) calculations, highlighting the role of hydrogen species—H^+^ and H^−^ pairs—in opening the unsaturated furan ring. The findings suggest that this selective hydrogenolysis follows an S_N_2 mechanism, with H^−^ acting as the nucleophile. Furthermore, systematic characterization and control experiments revealed the synergy between Pt nanoparticles (NPs) and Pt^*δ*+^⋯O^2−^ Lewis acid–base units, as well as the role of Fe in facilitating the ring-opening of furan compounds.

## Results and discussion

### Catalyst characterizations

**Fig. 1:**
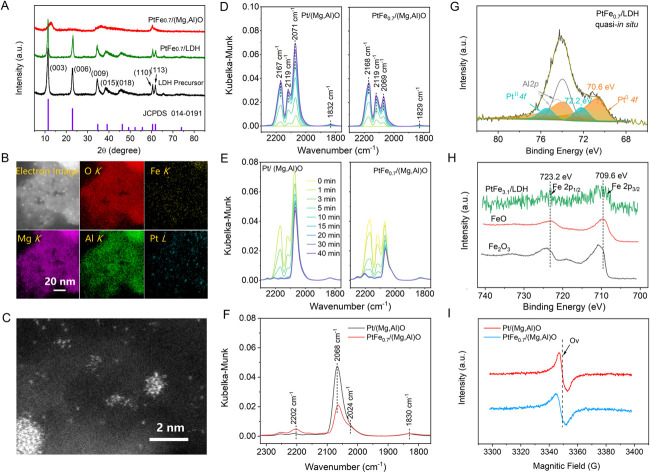
(A) XRD patterns of LDH precursor as well as PtFe_0.7_/(Mg,Al)O catalyst before and after water treatment. (B) HAADF-STEM image and elemental distribution mappings of PtFe_0.7_/(Mg,Al)O. (C) Aberration-corrected HAADF-STEM image of PtFe_0.7_/(Mg,Al)O. (D and E) The dependence of DRIFTS spectra for CO adsorption (D) and desorption (E) on time over Pt/(Mg,Al)O and PtFe_0.7_/(Mg,Al)O. (F) Comparison of DRIFTS spectra over Pt/(Mg,Al)O and PtFe_0.7_/(Mg,Al)O after CO adsorption and followed by a Ar flow of 40 min. (G) Quasi-*in situ* Pt 4f XPS spectra of PtFe_0.7_/LDH. (H) Quasi-*in situ* Fe 2p XPS spectra of PtFe_3.1_/LDH and FeO_*x*_ references. (I) EPR spectra of Pt/(Mg,Al)O and PtFe_0.7_/(Mg,Al)O.

The catalysts were synthesized using Mg,Al-LDH as the support precursor. The crystal phase of LDH is identified as Mg_6_Al_2_(OH)_18_·4.5H_2_O (PDF#35-0965) (Fig. 1A), and it exhibits irregular nanosheet morphology, as shown from the scanning electron microscopy (SEM) images (Fig. S1†). After calcination and H_2_ reduction treatment, the LDH converted to mixed Mg/Al oxide [(Mg,Al)O]. Catalysts with different Fe/Pt atomic ratio were prepared and the Fe/Pt atomic ratios were determined by inductively coupled plasma optical emission spectroscopy (ICP-OES), as shown in Table S1.† We denote the as-prepared catalyst PtFe_*x*_/(Mg,Al)O (*x* = Fe/Pt atomic ratio), which can revert to PtFe_*x*_/LDH under hydration conditions owing to the “memory effect”,^23^ as confirmed by the X-ray diffraction (XRD) pattern (Fig. 1A). High-angle annular dark field scanning transmission electron microscopy (HAADF-STEM) images and elemental distribution mappings of the PtFe_0.7_/(Mg,Al)O demonstrate the uniform dispersion of Pt and Fe on the support (Fig. 1B). Pt in Pt/(Mg,Al)O is found to exist in both NPs and atomically dispersed states, as observed through aberration-corrected HAADF-STEM (Fig. S2†). The PtFe_0.7_/(Mg,Al)O exhibits a similar morphology to Pt/(Mg,Al)O (Fig. 1C), indicating that the addition of Fe does not alter or influence the state of Pt. Scanning transmission electron microscopy with energy dispersive X-ray spectroscopy (STEM-EDX) line-scanning analysis of PtFe_0.7_/(Mg,Al)O demonstrates the presence of Fe deposition on the Pt surface (Fig. S3†).^12^

Diffuse reflectance infrared Fourier transform spectroscopy (DRIFTS) was conducted over Pt/(Mg,Al)O and PtFe_0.7_/(Mg,Al)O using CO as the probe to further explore the structure of the catalysts (Fig. 1D–F). As seen in Fig. 1D, the doublet peaks at approximately 2167 cm^−1^ and 2119 cm^−1^ are attributed to CO gas.^24,25^ Similar features of CO adsorption bands are also observed in samples without Pt, such as Fe/(Mg,Al)O and (Mg,Al)O (Fig. S4†). The band at around 2070 cm^−1^ is assigned to CO linearly adsorbed on Pt metal sites, while the shoulder at approximately 2024 cm^−1^ corresponds to CO adsorbed on lower-coordination Pt metal sites.^26–28^ The decrease in intensity of the CO adsorption band at 2070 cm^−1^ indicates a significant reduction in the exposed Pt metal sites in PtFe_0.7_/(Mg,Al)O compared with Pt/(Mg,Al)O. This may be attributed to the Fe oxide/hydroxide species deposited on the surface of Pt nanoparticles, which obscure partial active sites of Pt,^29^ which aligns with the STEM-EDX line-scanning results (Fig. S3†). The weak peak at around 1830 cm^−1^ is assigned to bridge-bonded CO adsorption on Pt. In DRIFTS spectra of CO desorption (Fig. 1E and F), the peak at 2202 cm^−1^ is associated with CO bound to cationic Pt sites,^24,30^ which is more prominent in PtFe_0.7_/(Mg,Al)O than in Pt/(Mg,Al)O.

Quasi-*in situ* X-ray photoelectron spectroscopy (XPS) analysis of PtFe_*x*_/LDH (the hydrothermally treated PtFe_*x*_/(Mg,Al)O samples) was further conducted in an anaerobic environment. Pt 4f spectrum clearly demonstrates that both Pt^0^ and Pt^2+^ are present on the surface of these catalysts (Fig. 1G).^31^ Compared with Pt/LDH, the binding energies (BE) of Pt^0^ in PtFe_*x*_/LDH exhibit a slight positive shift (Fig. S5†), indicating a lower electron density of surface Pt. This phenomenon could be attributed to electron transfer from Pt clusters to FeO_*x*_*via* Pt–O–Fe bonding.^29^ Notably, the oxidation state of Pt in PtFe_0.7_/LDH aligns with that observed in PtFe_0.7_/(Mg,Al)O sample (Fig. S6†). Additionally, the valence state of Fe is +2 under the anaerobic environment (Fig. 1H) but +3 in air (Fig. S7†). X-ray absorption spectroscopy (XAS) were employed to analyze the local electronic and geometric structures of PtFe_*x*_/(Mg, Al)O. The Pt L_3_-edge extended X-ray absorption fine structure (EXAFS) spectra of PtFe_*x*_/(Mg,Al)O exhibits two peaks at 2.0 and 2.7 Å, corresponding to the Pt–O and Pt–Pt bonds, respectively (Fig. S8, S9, and Table S2†), further affirming the co-existence of Pt single sites and clusters in PtFe_*x*_/(Mg,Al)O. The Fe K-edge EXAFS spectra of PtFe_*x*_/(Mg,Al)O (Fig. S10, S11 and Table S2†) also provide evidence for the absence of a Pt–Fe bond, confirming that no Pt–Fe alloy is formed.^32,33^ Electron paramagnetic resonance (EPR) spectroscopy were employed to characterize the paramagnetic centers of the catalysts. EPR signals of oxygen vacancies (O_v_) were observed at 3350 G in our catalysts (Fig. 1I).

### Hydrogenolysis of furanic compounds

The hydrogenolysis reaction was performed using FA as the substrate to investigate the selective cleavage of Csp^2^–O bond in furan ring over PtFe_*x*_/(Mg,Al)O catalysts. As aforementioned, under hydration conditions, (Mg,Al)O can be converted into LDH (Fig. 1A). Consequently, we refer to the catalysts as PtFe_*x*_/LDH during the reaction. The reaction was conducted under a H_2_ pressure of 1.0 MPa in water at 150 °C. As seen in Table 1, FA achieves a complete conversion within 5.5 h, with selectivity toward 1,2-pentanediol (1,2-PeD) and diols of 82.0% and 90.9%, respectively, marking one of the most promising results to date (Table S3†). Compared with the PtFe_0.7_/LDH catalyst, Pt/LDH exhibits similar activity but lower selectivity towards diols, while Fe/LDH exhibits only 1.4% FA conversion. This suggests that Pt is the primary active component for FA conversion, and Fe enhances the selectivity towards ring-opening products. This is because Fe reduces the electron density on the Pt surface (Fig. S5†),^31^ thereby improving the selectivity for C–O bond hydrogenolysis.

**Table 1:** Catalytic performance of various catalysts for FA hydrogenolysis^a^

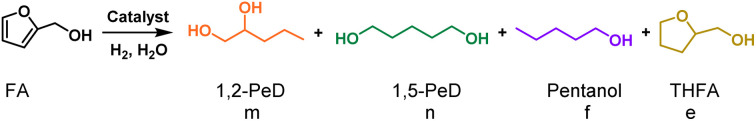						
Catalyst	Conv. (%)	Selectivity (%)				
		*m*	*n*	*f*	*e*	*m* + *n*
PtFe_0.7_/LDH	100	82.0	8.9	4.8	2.3	90.9
Pt/LDH	100	70.1	9.1	1.5	16.3	79.2
Fe/LDH	1.4	—	—	—	—	—
PtCo_0.8_/LDH	100	60.8	7.5	14.0	13.2	68.3
PtNi_0.7_/LDH	100	17.0	10.4	1.0	69.3	27.4
PtMn_0.7_/LDH	12.1	28.2	15.0	2.9	30.5	43.2
PtFe_0.7_/LDH^b^	59.1	73.8	18.2	6.3	1.7	92
PtFe_0.7_/LDH^c^	12.6	37.7	18.5	3.2	24.0	56.2

a Reaction conditions: 80 μL FA, 30 mg catalyst, 2.0 mL water, 1.0 MPa H_2_, 150 °C, 5.5 h. Pentanol including 1-pentanol and 2-pentanol.

b The solvent was 2.0 mL mixture of ethanol and water (v/v = 1/1).

c The solvent was 2.0 mL ethanol.

**Fig. 2:**
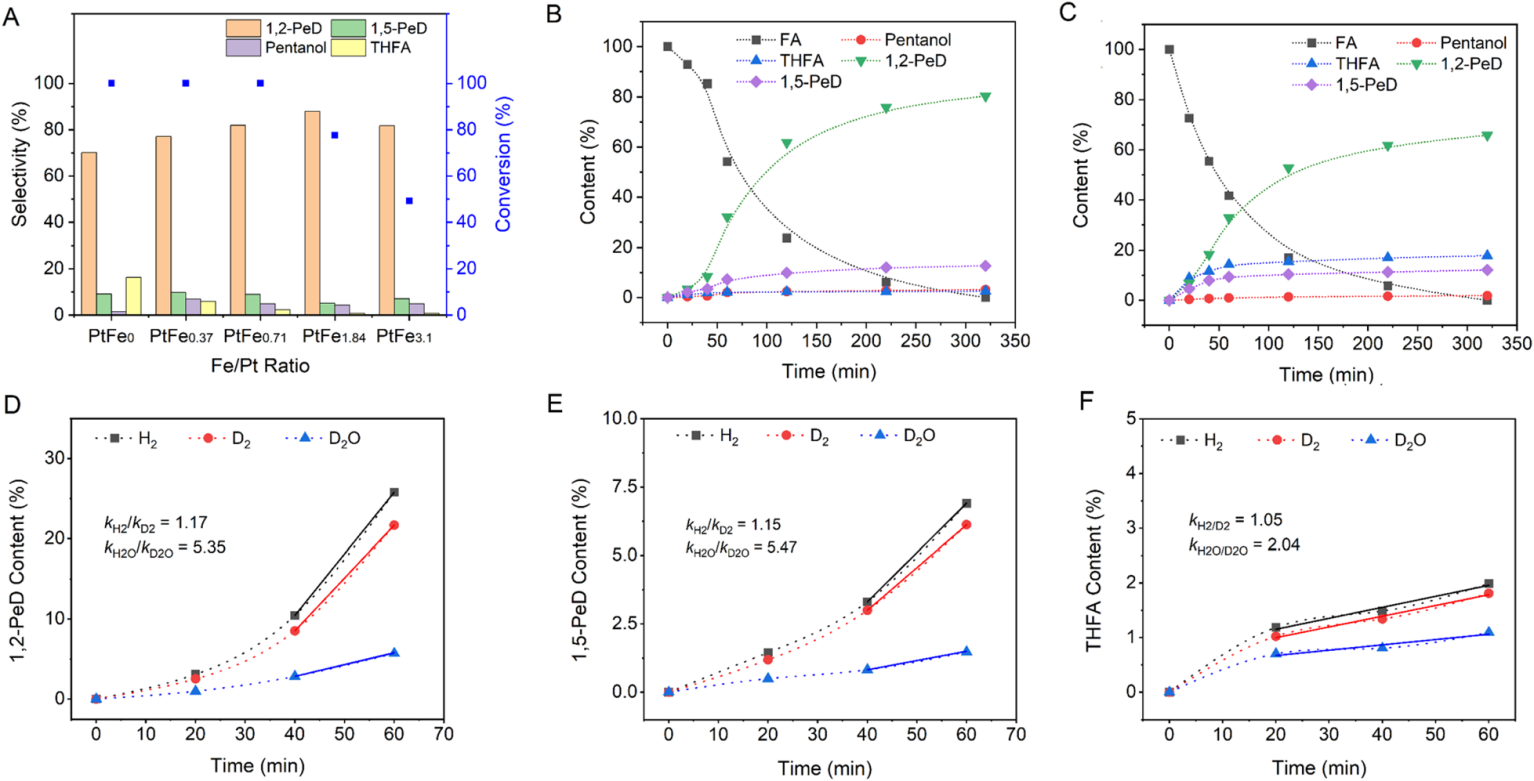
(A) FA hydrogenolysis over various PtFe_*x*_/LDH catalysts. (B) Plot of content of different species *vs.* time over PtFe_0.7_/LDH catalyst. (C) Plot of content of different species *vs.* time over Pt/LDH catalyst. (D–F) Primary isotope effect for the hydrogenolysis of FA to 1,2-PeD (D), 1,5-PeD (E) and THFA (F). The reaction conditions in A–C: 80 μL FA, 30 mg catalyst, 2.0 mL water, 1.0 MPa H_2_, 150 °C. The reaction conditions in D–F: 80 μL FA, 30 mg catalyst, 2.0 mL H_2_O (or D_2_O), 1.0 MPa H_2_ (or D_2_), 150 °C.

Furthermore, when Fe in PtFe_0.7_/LDH is substituted with other transition metals including Co, Ni and Mn, the conversion of FA or the selectivity of diols decreases. Specifically, the conversion of FA over PtMn_0.7_/LDH is only 12.1%, while the selectivity of diols is only 27.4% over PtNi_0.7_/LDH. The influence of the Fe/Pt ratio on the catalytic performance was further investigated (Fig. 2A). With increasing Fe content in PtFe_*x*_/(Mg,Al)O, the formation of the ring hydrogenation product tetrahydrofurfuryl alcohol (THFA) is suppressed, leading to increased selectivity toward 1,2-PeD. However, with further increasing Fe content, the conversion of FA noticeably decreases, indicating that the excessive Fe may reduce the activity of the catalyst. Recycling experiments were conducted to assess the stability of the PtFe_0.7_/LDH catalyst.^34^ The selectivity and conversion results indicate that the catalyst remains relatively stable over three cycles (Fig. S12).†

Fig. 2B and C illustrates the time-dependent FA conversion over Pt/LDH and PtFe_0.7_/LDH. 1,2-PeD remains the dominant product consistently over Pt/LDH and PtFe_0.7_/LDH. Notably, the cyclic hydrogenation product THFA is significantly inhibited by Fe introduction. This effect could be attributed to the reduced electron density of Pt nanoparticles (Fig. S5)† in the presence of Fe, which hinders the adsorption of C=C bonds and thereby suppresses ring hydrogenation.^29,31^ Kinetic isotope effect (KIE) experiments, which compare the reaction rates using H_2_ and D_2_, or H_2_O and D_2_O, were conducted by replacing hydrogen (H) with deuterium (D) either in water (KIE_H_2_O/D_2_O_ = *k*_H_2_O_/*k*_D_2_O_) or in H_2_ (KIE_H_2_/D_2__ = *k*_H_2__/*k*_D_2__) (Fig. 2D–F). The apparent KIE_H_2_/D_2__ value is approximately unity in both ring-opening and ring-hydrogenation reactions. Gas chromatography-mass spectrometer (GC-MS) (Fig. S13 and S14†) and nuclear magnetic resonance (NMR) (Fig. S15†) spectra indicate that D is distributed across almost all carbon atoms of the products. Considering the ∼16 : 1 total H/D ratio in the system, exchange between H and D likely occurs during hydrogen spillover (see below) before hydrogenation. The apparent KIE_H_2_O/D_2_O_ values for THFA production and the ring-opening reaction are noticeably different, with values of ∼2 and >5, respectively. The higher KIE_H_2_O/D_2_O_ value for the ring-opening reaction suggests that it is more likely to involve a high-barrier hydrogen transfer process than THFA production.

Hydrogenolysis reactions of other furanic compounds, including furan, furfural (FFR) and 5-hydroxymethylfurfural (HMF), were also conducted over the PtFe_0.7_/LDH catalyst. As anticipated, high selectivity towards ring-opened alcohols was achieved (Fig. S16–S19 and Table S4†). Furanic compounds with oxygenated side chains, such as FA, FFR and HMF, exhibit higher selectivity toward ring-opened products, benefiting from the oxygenated side chain preferentially absorbing on the catalyst surface. These catalytic results confirm that PtFe_0.7_/LDH is indeed a highly active and selective catalyst for the hydrogenolysis of various furanic compounds to polyols. Particularly, it is observed that FFR is fully converted into FA over PtFe_0.7_/LDH within 30 min (Fig. S20†), demonstrating the high activity of hydrogen species for the hydrogenation of polar C=O bond.

### Hydrogen spillover and Pt–hydrides

**Fig. 3:**
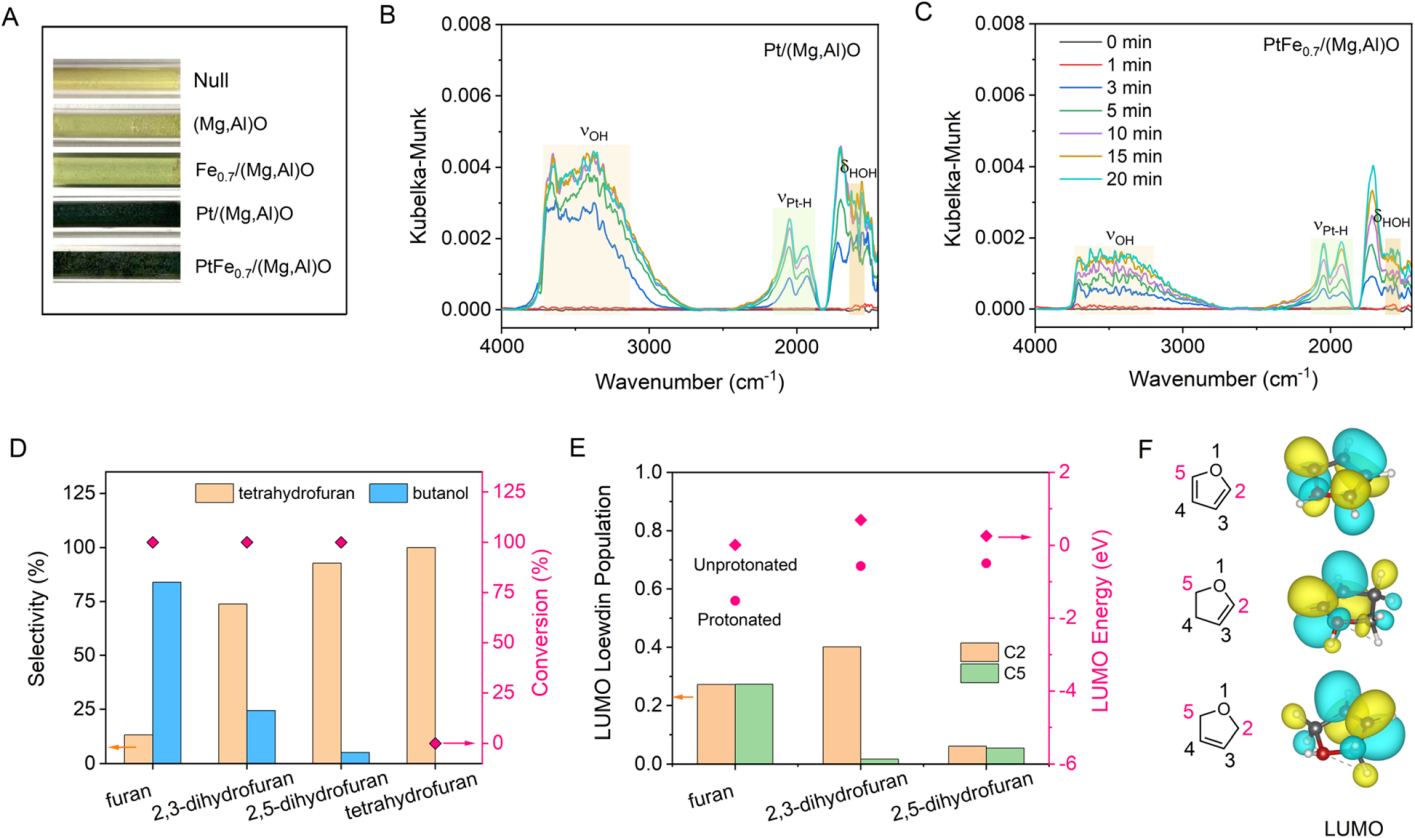
(A) Photographs of samples before and after treatment with H_2_ at 30 °C for 10 min. (B) DRIFTS spectra in H_2_ over Pt/(Mg,Al)O and PtFe_0.7_/(Mg, Al) O (C) catalysts. (D) Hydrogenolysis of various furanic substrates with different double bonds over PtFe_0.7_/(Mg,Al)O catalyst. Reaction conditions: 80 μL furanic compounds, 30 mg catalyst, 2.0 mL water, 1.0 MPa H_2_, 150 °C, 5.5 h. (E) The LUMO Löwdin populations of C2 and C5 in protonated furanic compounds and LUMO energy in protonated and unprotonated furanic compounds. (F) LUMOs of different furanic compounds, contoured at 0.029 a.u.

It has been established that H_2_ activation plays a crucial role in determining the selectivity of the catalysts for hydrogenation/hydrogenolysis.^15^ To distinguish H_2_ dissociation on Pt NPs or on Pt single sites, CO poisoning experiments were conducted over Pt/LDH and PtFe_0.7_/LDH catalysts. Upon the addition of 0.2 MPa CO, the conversion of FA decreases to 1.2% and 8% over Pt/LDH and PtFe_0.7_/LDH, respectively (Fig. S21†). Given that single metal sites exhibit higher CO tolerance compared with metal NPs,^35^ the reduced activity of catalysts can be attributed to the poisoned Pt NPs hindering H_2_ dissociation. Therefore, it can be speculated that H_2_ molecules are dissociated into H atoms on Pt NPs, which may subsequently spill over to the LDH support and Pt single atom sites.^36^ H_2_ spillover experiments were conducted by the reduction of WO_3_. The color of WO_3_ sample changed from yellow to black after being mixed with Pt/(Mg,Al)O or PtFe_*x*_/(Mg,Al)O treated with hydrogen, demonstrating the occurrence of H_2_ spillover (Fig. 3A). This is because H atom can readily react with yellow WO_3_ to form black H_*x*_WO_3_ when spillover occurs.^37^ The presence of oxygen vacancies on the (Mg,Al)O support may facilitate the hydrogen spillover process.^38,39^

An *in situ* diffuse reflection infrared Fourier transform spectroscopy (DRIFTS) study in H_2_ over Pt/(Mg,Al)O and PtFe_0.7_/(Mg,Al)O catalysts was conducted to analyze the surface species (Fig. 3B and C). The bands observed in the range of 2800∼3700 cm^−1^ and around 1600 cm^−1^ are assigned to hydroxyl stretching vibrations and water bending vibrations, respectively, which derives from the reduction of the supported PtO_*x*_ phase.^40,41^ It seems that the addition of Fe reduces the intensity of the hydroxyl and water peaks. According to the previous reports,^41,42^ the peaks at ∼2050 cm^−1^ and ∼1950 cm^−1^ are related to strongly adsorbed linear Pt–H species. The difference between these two strongly adsorbed species might be related to the type of environment around the hydride. It has been proven that under negative fields, the stretching frequency of Pt–H can shift to a lower frequency.^43^ As shown in Fig. 3B and C, compared with Pt/(Mg,Al)O, PtFe_0.7_/(Mg,Al)O exhibits a higher ratio of low-frequency Pt–H to high-frequency Pt–H, indicating that the addition of Fe promotes the generation of more negatively charged Pt–H species.

From the experimental observations, we propose that H_2_ molecules first undergo barrierless homolysis activation on metallic Pt NPs of PtFe_*x*_/(Mg,Al)O catalysts, forming neutral active H* species. In the presence of Pt^*δ*+^⋯O^2−^ Lewis acid–base sites, these neutral active H* species can then migrate either to Pt single sites, producing Pt–H^*δ*−^ species, or interact with oxygen species, producing O–H^+^ species.^44^ Therefore, Pt–H^*δ*−^ and O–H^+^ likely function as active species for the ring-opening reaction. Low conversion and diols selectivity are observed when the reaction is conducted in ethanol or a water/ethanol mixture (Table 1), suggesting that water is crucial for the hydrogenation/hydrogenolysis of FA, likely due to its role in hydrogen spillover and proton transfer.^18,45–49^

### Mechanistic analysis

To further clarify the reaction mechanism, the hydrogenolysis reaction of furan rings with different double bonds were conducted (Fig. 3D). The resulting order of ring-opening selectivity is furan > 2,3-dihydrofuran > 2,5-dihydrofuran > tetrahydrofuran = 0. DFT calculations show that the lowest unoccupied molecular orbital (LUMO) contains a higher contribution from C2 p-orbitals as well as significant C–O π antibonding character when a double bond is adjacent to the oxygen of the furan ring (Fig. 3E and F). This makes the carbon atom (C2) next to the oxygen more susceptible to nucleophilic attack by the hydride, resulting in the cleavage of the Csp^2^–O bond through an S_N_2 reaction.

As mentioned above, a significant normal KIE is observed (KIE_H_2_O/D_2_O_ > 5) in the ring-opening reaction (Fig. 2D and E). Such a large H–D KIE in hydrogenation has also been observed on a Pd single atom catalyst (*k*_H_/*k*_D_ = 5.75),^17^ suggesting that the rate-determining step is proton transfer rather than the hydrogen atom or hydride transfer from the Pt–H^*δ*−^ motif. Otherwise, an inverse KIE would be observed as the force constant of Pt–H bond is smaller than that of C–H bond.^50–52^ By comparison, for the ring hydrogenation to THFA, a smaller KIE (KIE_H_2_O/D_2_O_ ∼2) is observed, which may be attributed to a weighted average of two pathways: one involving neutral hydrogen (H*) on the Pt nanoparticle surface, and the other involving polar hydrogen (H^*δ*−^ and H^+^). The former pathway is dominant due to the nonpolar nature of the C=C bond.^47^

**Fig. 4:**
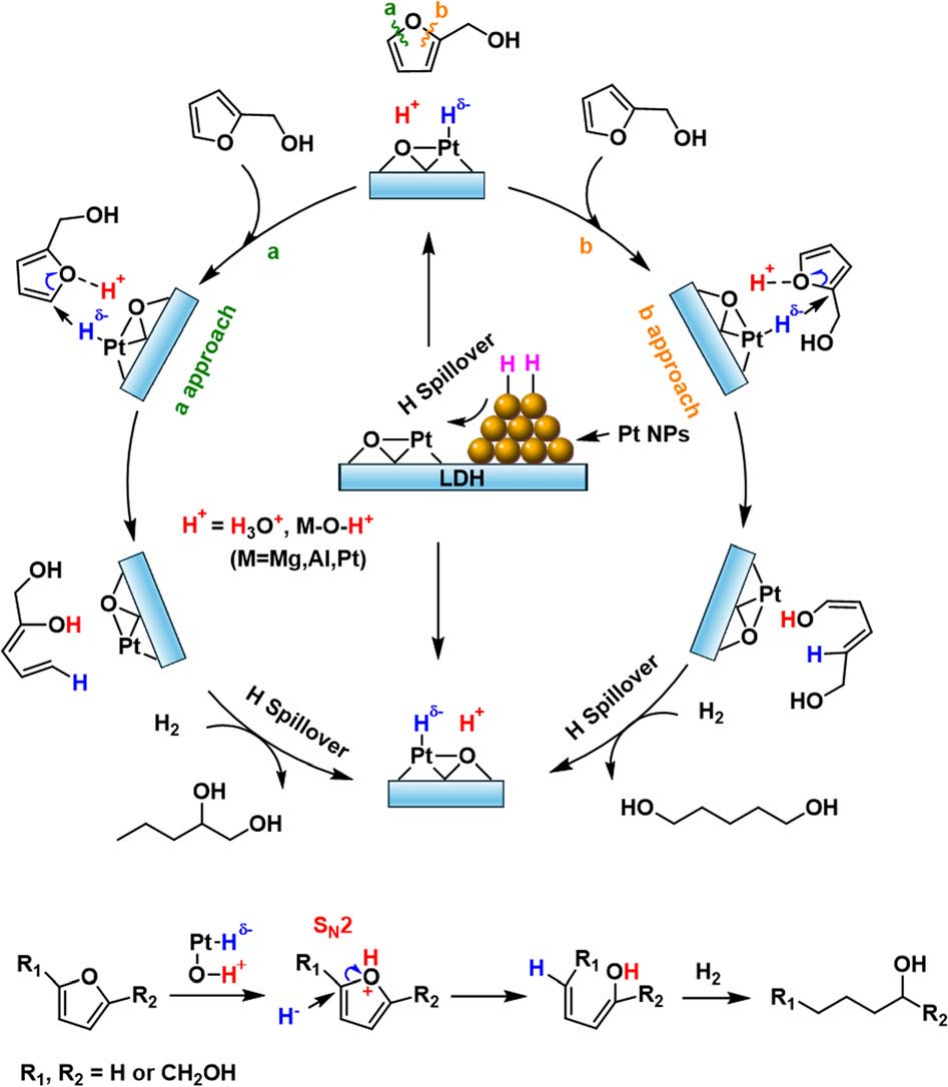
Proposed mechanism for the ring-opening of FA over PtFe_*x*_/LDH catalyst.

Based on the experimental and theoretical evidence, we propose the reaction mechanism for the ring-opening of FA over PtFe_*x*_/LDH as illustrated in Fig. 4. The FA hydrogenolysis starts from the barrierless homolysis activation of H_2_ on the Pt NPs, and then the active H* species migrate to the Pt single sites and the LDH support, producing Pt–H^*δ*−^ and O–H^+^ (proton) species at the Pt^*δ*+^⋯O^2−^ Lewis acid–base unit of PtFe_*x*_/LDH.^44^ It should be noted that in an aqueous solution, H^+^ may migrate to other oxygen atoms on the support or form hydrated protons during the transfer process. DFT calculations indicate a reduction in the LUMO energy after the furanic compounds are protonated (Fig. 3E and S22†). This suggests that the reaction is initiated by the proton combining with O on the furan ring, and subsequently H^*δ*−^ attacks the carbon atom of the Csp^2^–O bond in the furan ring, initiating an S_N_2 reaction that cleaves the furan ring. Upon completion of the ring-opening, the reaction intermediates form unsaturated alcohols. Subsequent hydrogenation of these unsaturated alcohols yields the final desired alcohol products.

Further examination of the DFT results reveals the Löwdin population of the LUMO on the carbon atom with oxygenated side chain is lower compared with that without such a chain (Fig. S22†), leading to H^*δ*−^ predominantly targeting the carbon atom of the Csp^2^–O bond furthest from the hydroxymethyl group. The steric hindrance caused by the hydroxymethyl group may be another reason for the higher selectivity of 1,2-PeD over 1,5-PeD.^53^ The addition of Fe enhances catalytic selectivity by facilitating the formation of Fe oxide/hydroxide species on the Pt surface, which inhibit the adsorption of vinyl groups and consequently suppress the hydrogenation of the furan ring.^29^

## Conclusions

In this work, we developed PtFe_*x*_/(Mg,Al)O [PtFe_*x*_/LDH under hydrothermal conditions] catalysts for the selective hydrogenolysis of the Csp^2^–O bond in the furan ring to polyols. Over 90% selectivity for diols or triols can be achieved at complete conversion of the furanic compounds using the PtFe_0.7_/LDH catalyst. During the reaction, H_2_ dissociates on Pt NPs, and active hydrogen atoms migrate to the Pt^*δ*+^⋯O^2−^ unit of PtFe_*x*_/LDH to produce Pt–H^*δ*−^ and O–H^+^ pairs *via* spillover. The resulting Pt–H^*δ*−^ and O–H^+^ serve as active species for cleaving the Csp^2^–O bond through S_N_2 reaction, facilitating the ring-opening of furanic compounds. The introduction of Fe inhibits the adsorption of vinyl groups on the Pt surface, thereby suppressing the hydrogenation of the furan ring. This study underscores the synergistic interplay among NPs, single metal sites and Lewis basic sites on the support in achieving the selective hydrogenation of furanic compounds. Additionally, the regioselectivity of ring-opening reaction are elucidated at the molecular level. We anticipate that these findings will provide valuable guidance for designing catalysts for other selective hydrogenation reactions.

## Author contributions

FFP: performed the experiments and analysis, as well as wrote the manuscript. BXH, XCK and BFC: funding acquisition, supervision and edited the manuscript. JKL: performed the computations, wrote the computational methodology, and participate in spectroscopic and mechanistic analysis. BZ: participate in DRIFT data analysis. RYZ, SJX and LLK: performed the EXAFS data fitting and analysis. Other people: participated in discussion.

## Conflicts of interest

There are no conflicts to declare.

## Supplementary Material

SC-015-D4SC05751A-s001

## Data Availability

The data supporting this article have been included as part of the ESI.†
