# Supplementary material for: Selective hydrogenolysis of the Csp2–O bond in the furan ring using hydride–proton pairs derived from hydrogen spillover
Source: Chem Sci. 2024 Oct 28;15(46):19283–9. doi: 10.1039/d4sc05751a (PMC11533051; doi:10.1039/d4sc05751a)
Supplement: SC-015-D4SC05751A-s001 [file SC-015-D4SC05751A-s001.pdf]

Supplementary Information (SI) for Chemical Science.
This journal is © The Royal Society of Chemistry 2024

Supplementary Material for

Selective Hydrogenolysis of the Csp^2–O Bond in the Furan Ring Using Hydride-Proton Pairs Derived from Hydrogen Spillover

Fangfang Peng,[a] Bin Zhang,[a] Runyao Zhao,[a,b] Shiqiang Liu,[a] Yuxuan Wu,[a,b] Shaojun Xu,[c] Luke L. Keenan,[d] Huizhen Liu,[a,b] Qingli Qian,[a,b] Tianbin Wu,[a] Haijun Yang,[a] Zhimin Liu,[a,b] Jikun Li,*[a,e] Bingfeng Chen,*[a] Xinchen Kang,*[a,b] Buxing Han*[a,b,f]

1. Chemicals

$H_2PtCl_6·6H_2O$ (>99%) was purchased from Alfa Aesar China Co., Ltd. $Fe(NO_3)_3·9H_2O$ (>99%), $Ni(NO_3)_2·6H_2O$ (>99%), $Co(NO_3)_2·6H_2O$ (>99%), $Mg(NO_3)_2·6H_2O$ (>99%), Al $(NO_3)_3·9H_2O$ (>99%), NaOH (>99%) and Na_2CO_3 (>99%) were obtained from Sinopharm Chemical Reagent Co., Ltd. Furan (stabilized with BHT), HMF (>98%), FFR (>99%) and FA (>98%) were provided by Beijing Innochem Science & Technology co., LTD. All the reagents were used without further purification.

2. Material synthesis

2.1 Synthesis of (Mg,Al)O

(Mg,Al)O with Mg/Al molar ratio of 3:1 was synthesized using co-precipitation method.[1] At first, 100 mL of salt solution [1.2 M $Mg(NO_3)_2·6H_2O$ + 0.4 M Al $(NO_3)_3·9H_2O$] and 100 mL of NaOH aqueous solution (6 M) were slowly introduced into 50 mL of Na_2CO_3 aqueous solution (0.8 M) under vigorous stirring (800 rpm) at 65 ^oC for 18 h. The final solid was separated, washed with deionized water for 5 times and dried in vacuo at 60 ^oC for 24 h.

2.2 Synthesis of PtFe$_x$/ (Mg,Al)O catalysts

The PtFe$_x$/(Mg,Al)O catalysts were synthesized by a wet impregnation method. First, 0.6 g of (Mg,Al)O support was dispersed into 10 mL of deionized water and vigorously stirred for 10 min. Then, 2 mL of mixture containing $H_2PtCl_6·6H_2O$ (40.0 mg) and desired amount of Fe $(NO_3)_3·9H_2O$ was slowly dropped into the suspension and stirred at 50 °C for 12 h. The solid was separated, washed with deionized water for 5 times and dried in vacuo at 60 ^oC for 24 h, and then calcinated at 300 ^oC for 3 h in air. Finally, the solid was reduced in H_2/Ar (10% H_2) at 300 ^oC for 3 h in a tube furnace and PtFe$_x$/(Mg,Al)O catalysts were obtained.

3. Characterizations

3.1 Material characterizations

SEM was conducted on HITACHI S-8020. HRTEM and HAADF-STEM as well as elemental distribution mappings were performed on a JEOL ARM200F. XRD analysis was performed on the X-ray diffraction (Model D/MAX2500, Rigaka) with Cu-Kα radiation (λ = 1.54056 Å) and the scattering range of 2θ was from 5° to 80°, with a scanning rate of 5° min^{-1}. X-ray photoelectron spectroscopy (XPS) was conducted on an X-ray photoelectron spectrometer (USA, Thermo Fischer, ESCALAB 250Xi) equipped with a monochromatized Al Kα excitation source (1486.8 eV), using C1s (284.8 eV) of adventitious carbon as the standard. Quasi-*in situ* XPS experiments were performed by loading the freshly reduced sample (under H_2/Ar flow at 300 °C for 30 min) into the XPS sample holder inside a glove box, followed by transferring it into an ultra-high-vacuum chamber for XPS measurements.[2] Prior to pre-reduced with H_2/Ar, the $PtFe_x$/(Mg,Al)O catalysts were hydrothermally treated at 65 °C for 24h. EPR spectra were recorded on Bruker ELEXSYS E500-T X-band spectrometer with an ER-4119HS high-sensitivity probe, microwave power of 0.2 mW, modulation amplitude of 0.2 mT and modulation frequency of 100 kHz. Spectra were acquired at 77 K using a vacuum insulated quartz liquid nitrogen immersion dewar inserted into the EPR resonator.

3.2 XAFS

Pt *L3*-edge, Fe *K*-edge XAFS experiments were carried out on the 1W1B and 4B9A beam line of the Beijing Synchrotron Radiation Facility. Data processing and analysis were performed following standard methods.[3] The E0 values of 11564 and 7112 eV were used to calibrate all data with respect to the first inflection point of the absorption *L*3-edge of Pt foil and K-edge of Fe foil. The backscattering amplitude and phase shift functions for specific atom pairs were calculated ab initio using the FEFF8 code. X-ray absorption data were analyzed using standard procedures, including pre- and post-edge background subtraction, normalization with respect to edge height, Fourier transformation and non-linear least-squares curve fitting. The normalized k^3-weighted EXAFS spectra, $k^3 \times (k)$, were Fourier transformed in a k range from 2.6 to 11.5 $Å^{-1}$, to evaluate the contribution of each bond pair to the Fourier transform peak. The experimental Fourier spectra were obtained by performing an inverse Fourier transformation with a Hanning window function with r between 1.2–3.0 Å. The S_0^2 (amplitude reduction factor) value was determined based on the fitting results of metal-foil (1.09 for Pt, 0.75 for Fe).

3.3 Hydrogen spillover detection by WO_3

The hydrogen spillover experiments were conducted according to the previous literature.[4] Initially, 1 g of WO_3 and 0.02 g of catalyst was mixed, and the mixture was placed in a glass reaction tube and held in place with silica wool before being placed into an oven. Then, 10% H_2/Ar mixture was flowed into the tube at the temperature of 30 °C for 10 min. The color change of the powder samples was observed and captured by a digital camera.

3.4 DRIFTS

3.4.1 DRIFTS in CO

The DRIFTS measurements were performed on Bruker v70 spectrometer. A Harrick DRIFTS cell was used with ZnSe windows. Prior to the experiment, the catalysts were pretreated with Ar at 150°C for 30 min. Following that, CO was flowed for 40 min. Upon the saturation of CO, Ar was flowed to remove

CO. The flow rate of Ar or CO was 100 mL min^{-1}. During the whole process, the IR spectra were recorded with a resolution was 4 cm^{-1}.

3.4.2 DRIFTS in H_2

The DRIFTS measurements were performed on Thermo Nicolet IS50 instrument with a Hg-Cd-Te detector. A Harrick DRIFTS cell was used with ZnSe windows. Prior to the experiment, the catalysts were pretreated with Ar at 30 °C for 10 min. Following that, 5% H_2/Ar mixture was flowed for 20 min. The flow rate of Ar or H_2/Ar was 100 mL min^{-1}. During the whole process, the IR spectra were recorded with a resolution of 8 cm^{-1}.

4. Catalytic reaction

4.1 Hydrogenolysis reaction

Hydrogenolysis of furanic compounds was carried out in a stainless-steel autoclave reactor with a PTFE liner (15 mL). In a typical experiment, 0.14 g (80μL) of furanic compound, 30 mg of catalyst and 2.0 mL of water were added to the reactor, which was heated to desired temperature. Then, 1.0 MPa H_2 was charged into the reactor and the stirrer was started with a stirring rate of 800 rpm. After a desired reaction time, the reactor was cooled down to room temperature. The mixture in the reactor was separated by a centrifuge. For the hydrogenolysis of furan, FFR and FA, the liquid mixture was diluted by ethanol and was then quantitatively analyzed by GC (Agilent 7890A) and GC-MS (Agilent 7890B/5977A). For the hydrogenolysis of HMF, the liquid mixture was analyzed and quantified by ^{1}H-NMR (Bruker 300 and NEO 700), GC-MS (Agilent 7890B/5977A), GC (Agilent 7890A) and LC (LC-10AT, SHIMADZU). The material balance for all runs is higher than 95%.
The mass balance, conversion, selectivity and yield were counted by following equations:

$$Mass\ balance(\%) = \frac{Carbon\ in\ detactable\ products\ (mol)}{Furanic\ compound\ converted\ (mol)} \times 100\%$$

$$Selectivity(\%) = \frac{product\ generated}{substrate\ added - substrate\ left} \times 100\%$$

$$Conversion(\%) = \frac{substrate\ charged - substrate\ left}{substrate\ added} \times 100\%$$

$$Yield\ of\ product(\%) = \frac{product\ generated}{substrate\ added} \times 100\%$$

5. Isotopic Labeling Experiments

Isotope-labeling kinetic studies were conducted in a batch reactor under conditions similar to regular catalytic hydrogenation reactions, with the exception of substituting D_2 for H_2 or D_2O for H_2O. For testing $KIE_{H2/D2}$, 30 mg of Pt catalyst and 80 μL of FA were added into 2.0 mL of H_2O, and the reaction was performed at 150 °C with 1 Mpa of D_2. For calculating $KIE_{H2O/D2O}$, 30 mg of Pt catalyst and 80 μL of FA were added into 2.0 mL of D_2O, and the reaction was performed at 150 °C with 1 Mpa of H_2. The

liquid products containing deuterated molecules were analyzed by GC−MS and NMR.

6. DFT calculations

The geometries of furanic compounds were optimized with Gaussian 16 at the B3LYP/Def2TZVP level,[5] with the SMD solvent model (water as solvent)[6] and Grimme's D3 dispersion correction.[7] LUMO energy calculation and Löwdin population analysis on C2 and C5 were performed with ORCA 5.0.4 at the same level.[8]

7. Supplementary Figures

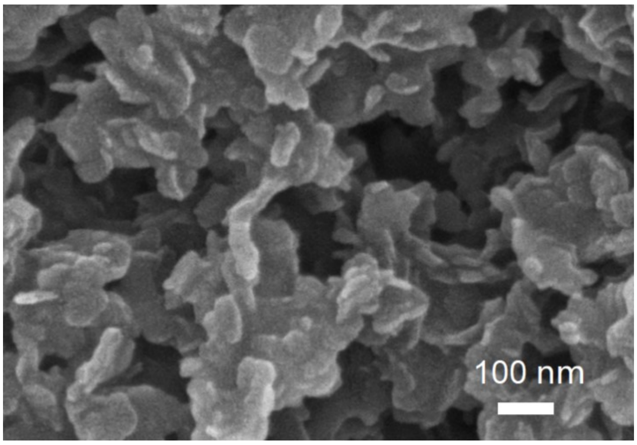

Figure S1 The SEM image of DLH precursor.

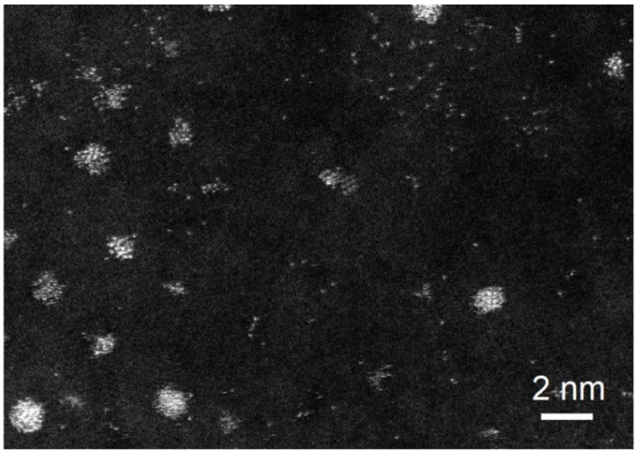

Figure S2 Aberration-corrected HAADF-STEM image of Pt/(Mg,Al)O catalyst.

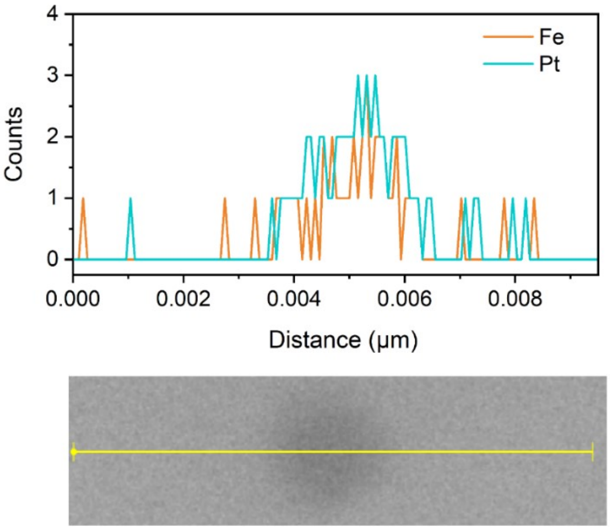

Figure S3 STEM-EDX line-scanning of the $PtFe_{0.7}$/(Mg,Al)O catalyst.

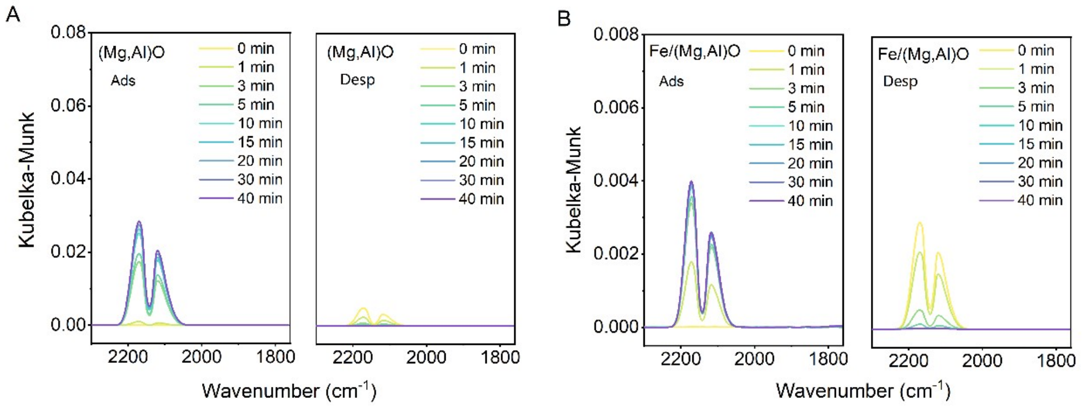

Figure S4 (A–B) DRIFTS spectra of CO adsorption and desorption over (Mg,Al)O **(A)** and Fe/(Mg,Al)O **(B)**.

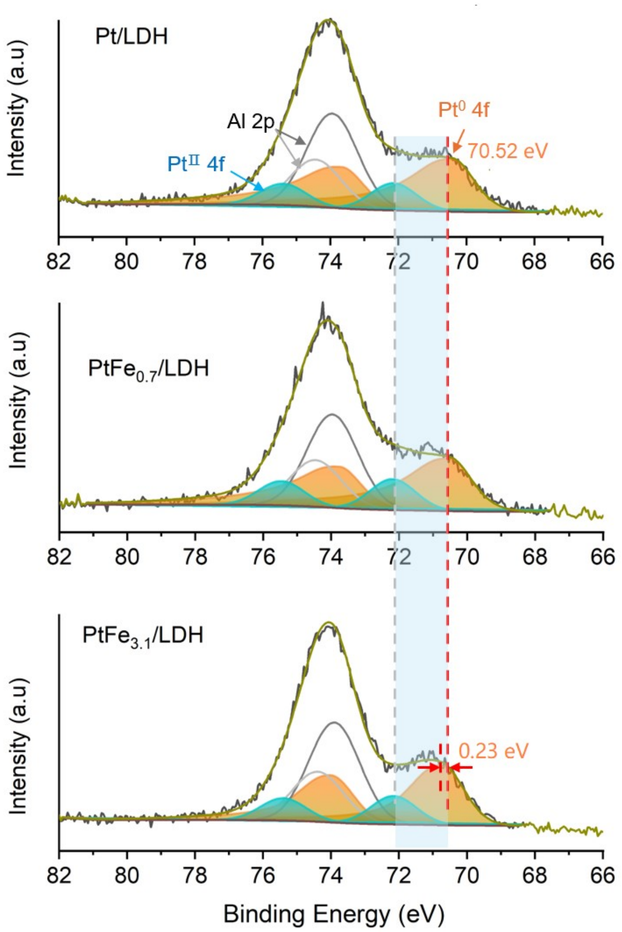

Figure S5 Quasi-*in situ* Pt *4f* XPS spectra of various PtFe$_x$/LDH (x=0, 0.7, 3.1) catalysts.

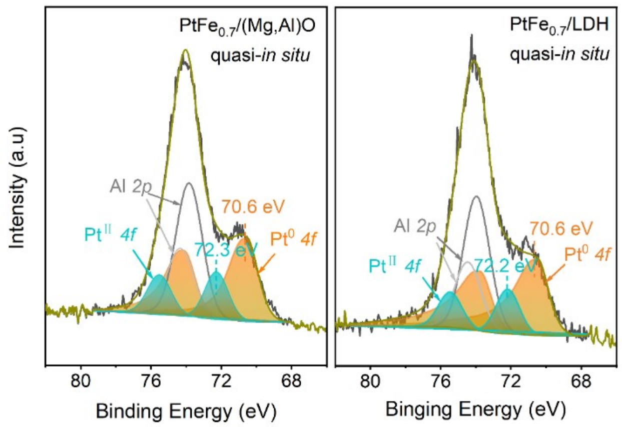

Figure S6 Quasi-*in situ* Pt *4f* XPS spectra of $PtFe_{0.7}$/(Mg,Al)O and $PtFe_{0.7}$/LDH.

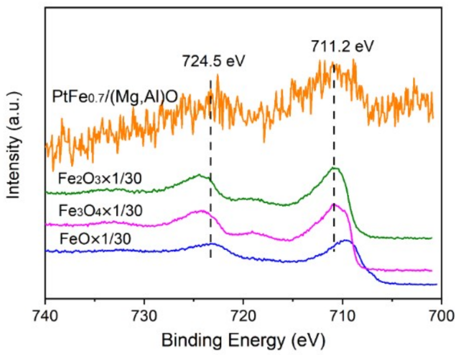

Figure S7 *Ex situ* Fe *3p* XPS spectra of $PtFe_{0.7}$/(Mg,Al)O and different FeO_x references.

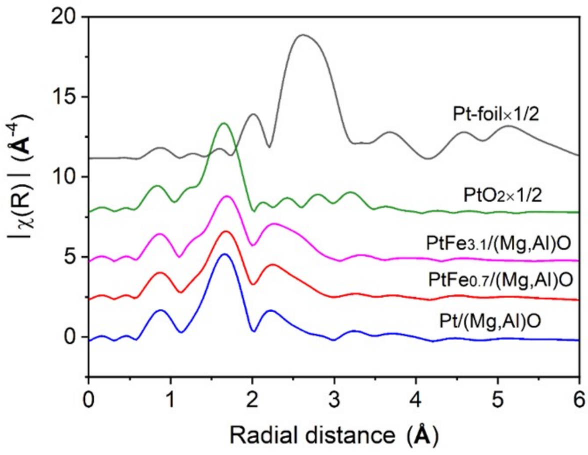

Figure S8 Fourier transforms of Pt *L*3-edge EXAFS spectra of $PtFe_x$/(Mg,Al)O, Pt foil and PtO_2 references.

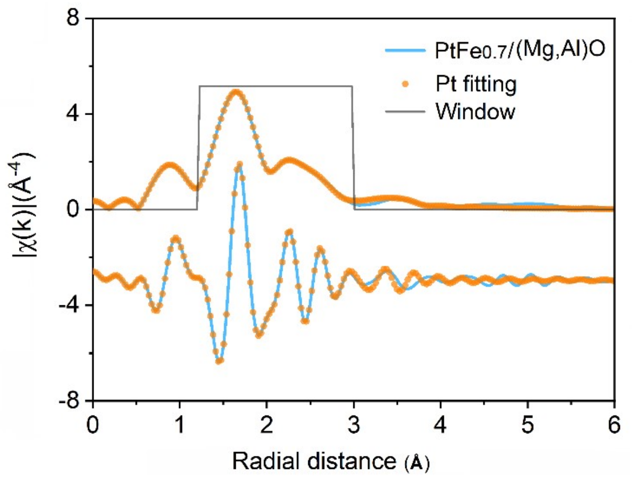

Figure S9 EXAFS R and K space fitting curves of Pt *L*-edge EXAFS spectra of $PtFe_{0.7}$/(Mg,Al)O.

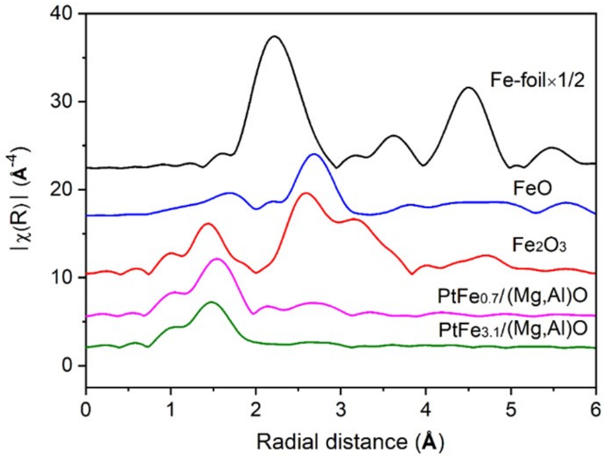

Figure S10 Fourier transforms of Fe *K*-edge EXAFS spectra of $PtFe_x$/(Mg,Al)O and different FeO_x references.

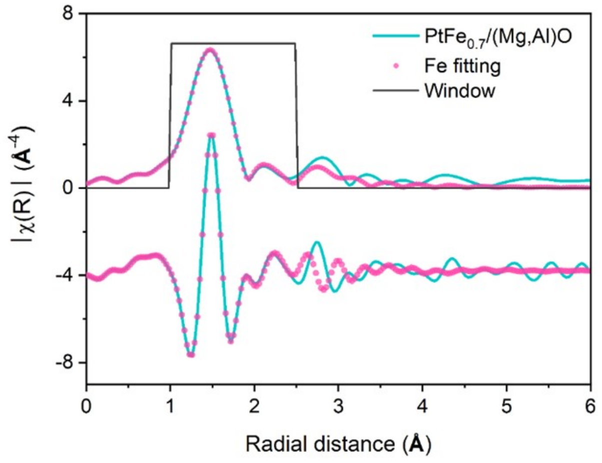

Figure S11 EXAFS R and K space fitting curves of Fe *K*-edge EXAFS spectra of $PtFe_{0.7}$/(Mg,Al)O.

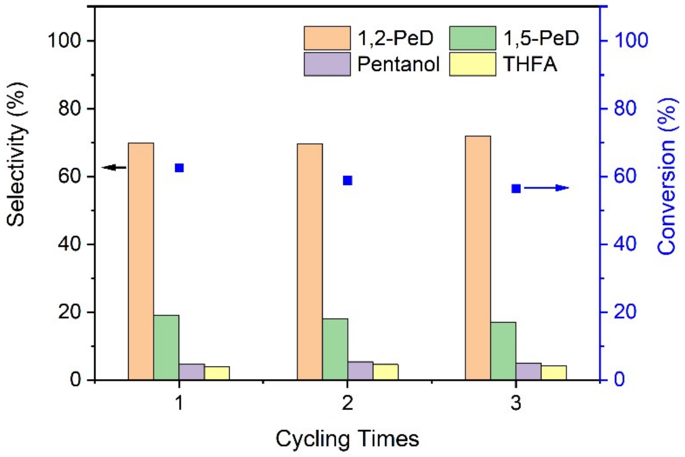

Figure S12 The cyclic stability of $PtFe_{0.7}$/LDH catalyst.

Reaction conditions: 0.22g FA, 70 mg of catalysts, 2.0 mL water, 1.5 MPa H_2, 150 °C, 1.0 h. After the reaction, the catalyst was washed three times with water and then dried under vacuum. Initially, two hydrogenolysis experiments were performed to compensate for any loss during the catalyst treatment process.

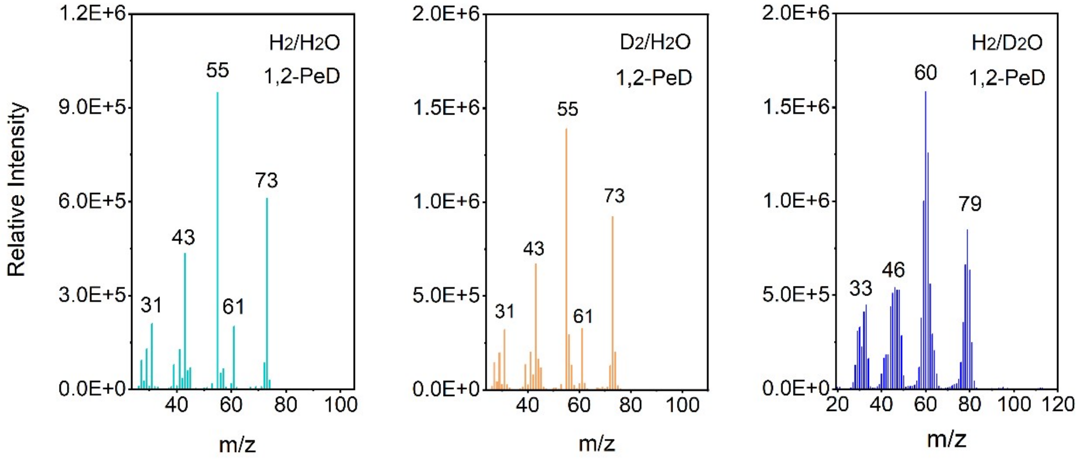

Figure S13 Mass spectra of 1,2-PeD obtained from FA hydrogenolysis using H_2/H_2O, D_2/H_2O, and H_2/D_2O. Reaction conditions: 80 μL FA, 30 mg catalyst, 2.0 mL H_2O (or D_2O), 1.0 MPa H_2 (or D_2), 150 °C.

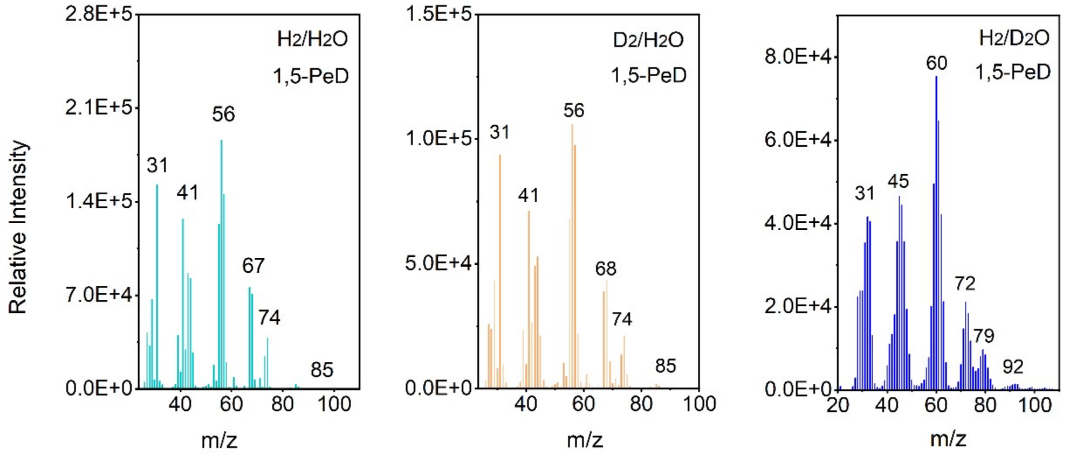

Figure S14 Mass spectra of 1,5-PeD obtained from FA hydrogenolysis using H_2/H_2O, D_2/H_2O and

H_2/D_2O. Reaction conditions: 80 μL FA, 30 mg catalyst, 2.0 mL H_2O (or D_2O), 1.0 MPa H_2 (or D_2), 150 °C.

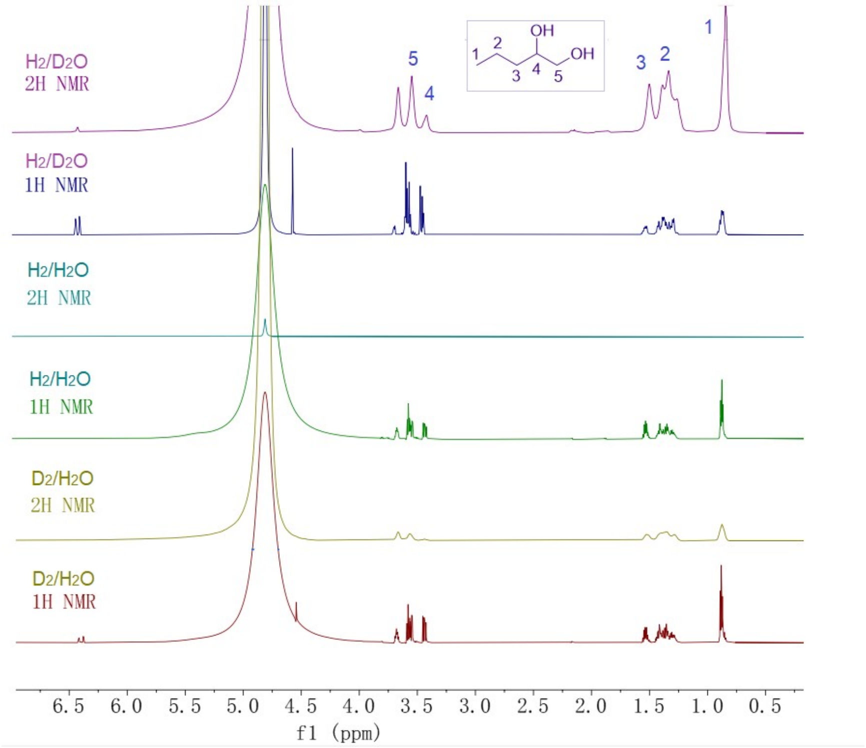

Figure S15 ^1H and ^2H NMR spectra after FA hydrogenolysis at different conditions for KIE study. During the NMR test, no deuterated reagent was added.

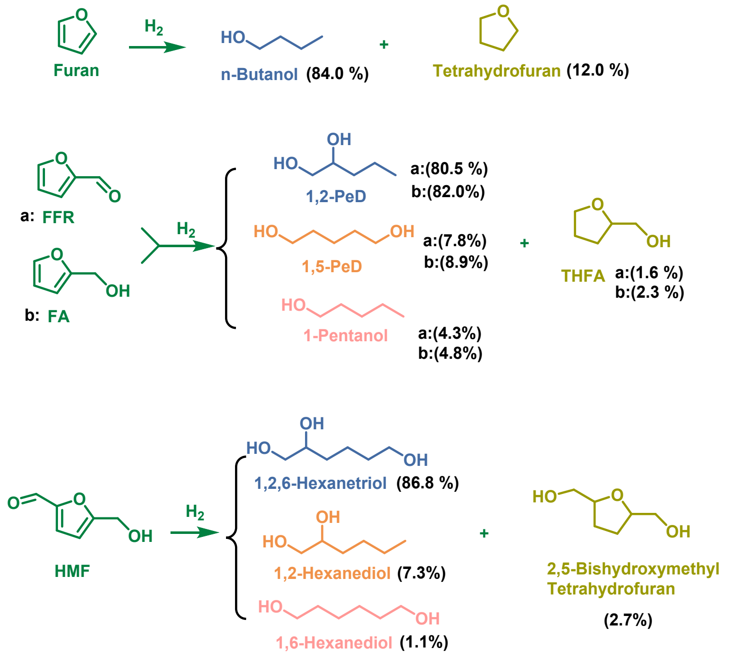

Figure S16 Hydrogenolysis of different furanic compounds over $PtFe_{0.7}/(Mg,Al)O$ catalyst. Reaction conditions: 80 μL FA, 30 mg catalyst, 2.0 mL water, 1.0 MPa H_2, 150 °C.

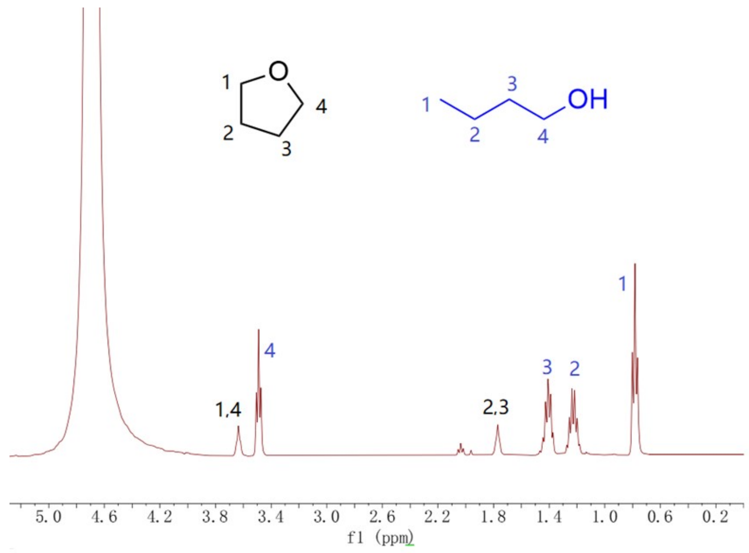

Figure S17 ^{1}H NMR spectra of the solution after hydrogenolysis of Furan.

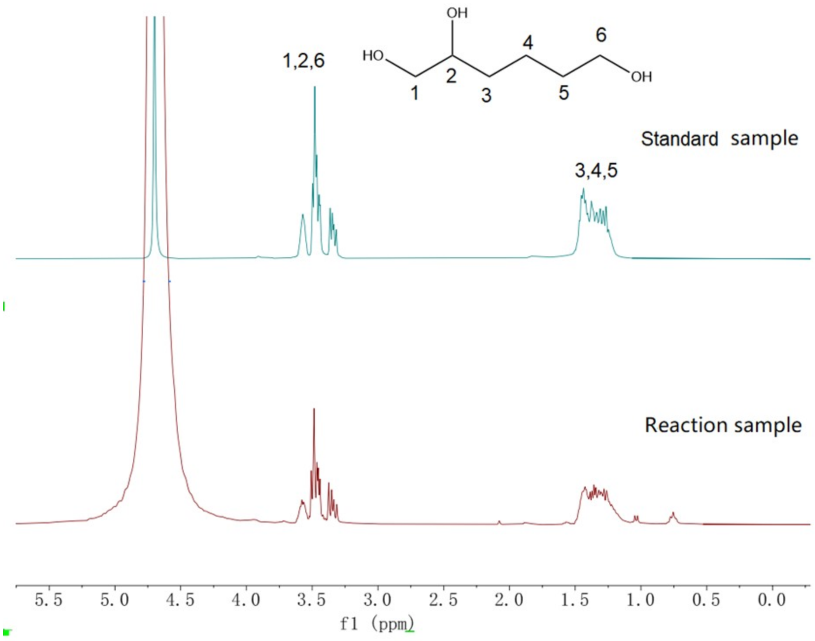

Figure S18 ^{1}H NMR spectra of 1,2,6-hexanetriol and the solution after hydrogenolysis of HMF.

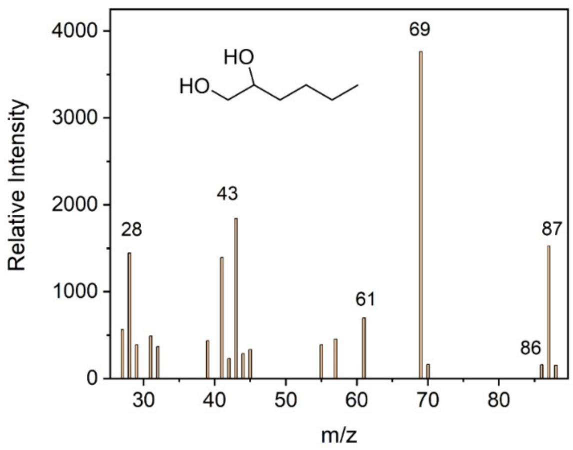

Figure S19 Mass spectra of 1,2-hexanediol obtained from HMF hydrogenolysis.

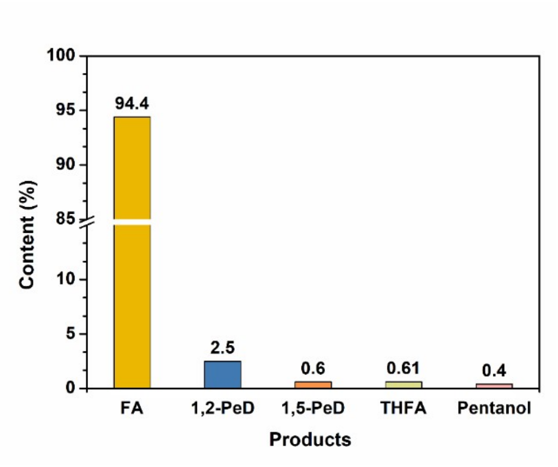

Figure S20 Product distribution for the FFR hydrogenolysis over $PtFe_{0.7}$/(Mg,Al)O after 30 min. Reaction conditions: 80 µL FA, 30 mg catalyst, 2.0 mL water, 1.0 MPa H_2, 150 °C, 0.5h.

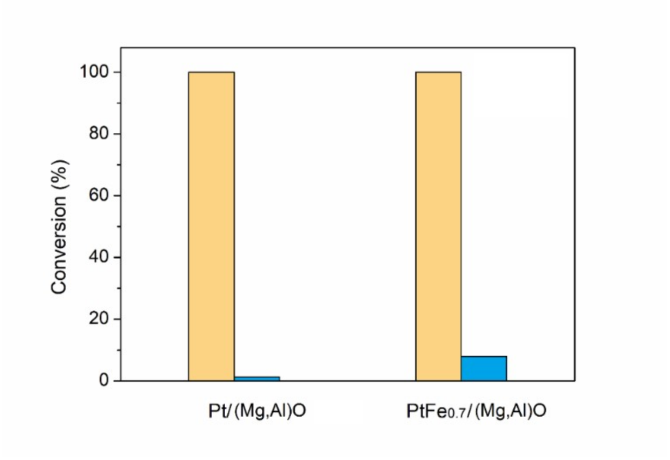

Figure S21 FA hydrogenolysis over $PtFe_{0.7}$/(Mg,Al)O in the absence (yellow) and presence (blue) of CO. Reaction conditions: 80 µL FA, 30 mg catalyst, 2.0 mL water, 1.0 MPa H_2 (or 1.0 MPa H_2+ 0.2 MPa CO), 150 °C, 0.5h.

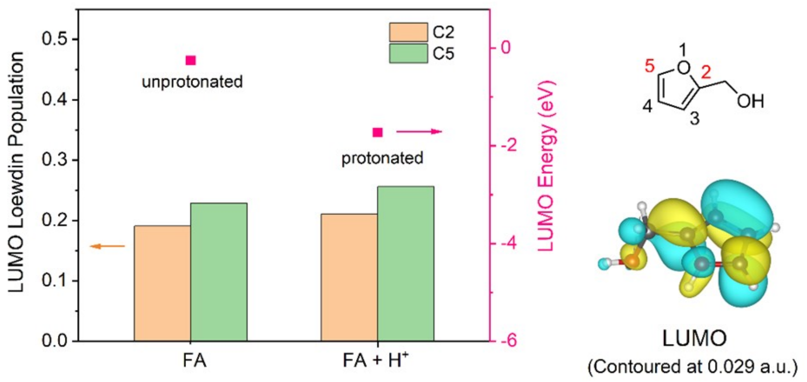

Figure S22 The LUMO Löwdin populations of C2 and C5 and LUMO energy in protonated and unprotonated of FA.

8. Supplementary Tables

Table S1 Actual metal loadings of PtM_x/(Mg,Al)O catalysts measured by ICP-OES.

Sample	Pt (wt%)	M (wt%)		M/Pt atomic ratio
Pt/(Mg,Al)O	2.12	/	/	0
$PtFe_{0.7}$/(Mg,Al)O	2.16	Fe	0.44	0.7
$PtFe_{1.8}$/(Mg,Al)O	1.99	Fe	1.05	1.8
$PtFe_{3.1}$/(Mg,Al)O	1.85	Fe	1.64	3.1
$PtNi_{0.7}$/(Mg,Al)O	2.02	Ni	0.42	0.7
$PtCo_{0.8}$/(Mg,Al)O	2.12	Co	0.52	0.8
$PtMn_{0.7}$/(Mg,Al)O	2.52	Mn	0.48	0.7

Table S2 EXAFS fitting parameters at the Pt L3-edge and Fe K-edge for various samples.

Sample	scatter	CN	*R* (Å)	ΔE_0 (eV)	S_0^2	σ^2	R factor
Pt/(Mg,Al)O	Pt–O	1.65±0.53	2.02±0.06	9.50±3.58	1.09	0.0003±0.004	0.017
	Pt–Pt	1.41±0.32	2.67±0.01	4.34±15.9	1.09	0.006±0.003	
Fe/(Mg,Al)O	Fe–O	4.44±0.75	2.05±0.09	-2.98±2.20	0.75	0.008±0.003	0.012
$PtFe_{0.7}$/(Mg,Al)O	Pt–O	1.38±0.64	2.02±0.05	8.42±5.52	1.09	0.001±0.006	0.019
	Pt–Pt	0.97±0.13	2.64±0.02	11.8±5.10	1.09	0.007±0.001	
	Fe–O	4.32±0.64	2.05±0.08	-2.00±1.93	0.75	0.006±0.002	0.010
$PtFe_{3.1}$/(Mg,Al)O	Pt–O	1.15±0.82	2.02±0.06	8.53±9.33	1.09	0.0006±0.009	0.019
	Pt–Pt	1.82±0.16	2.64±0.02	0.53±9.18	1.09	0.0086±0.002	
	Fe–O	3.90±0.20	2.05±0.09	-1.93±0.68	0.75	0.008±0.0009	0.001
Fe foil	Fe–Fe	8	2.52±0.05	4.75±1.58	0.75	0.005±0.001	0.009
Pt foil	Pt–Pt	12	2.77±0.01	7.53±0.42	1.09	0.004±0.0005	0.002
Fe_2O_3	Fe–Fe	4	2.89±0.06	-1.46±1.95	0.75	0.002±0.001	0.009
	Fe–O	6	2.09±0.08	-1.46±1.95	0.75	0.011±0.002	
PtO_2	Pt–O	6	2.01±0.01	9.56±0.75	1.09	0.002±0.001	0.013
	Pt–Pt	2	3.08±0.05	9.56±0.75	1.09	0.002±0.001	

R: bond distance; σ^2: Debye-Waller factors; ΔE_0: the inner potential correction. R factor: goodness of fit.

Table S3 Catalytic performances of various catalysts for the hydrogenolysis of FFR and FA.

Entry	Catalysts	Solvent	H_2 Pressure (MPa)	Substrate	Conv. (%)	Sel. (%) 1,2-PeD	1,5-PeD	THFA	Ref.
1	$PtFe_{0.7}/(Mg,Al)O$	H_2O	1.0	FA	100	82.0	8.9	2.3	This work
				FFR	100	80.5	7.8	1.6	
2	Pt/LDH	i-PrOH	3.0	FFR	>99	73	8	14	9
3	Ru-Sn/ZnO	i-PrOH	3.0	FFR	100	84.5	12.4	0	10
4	Rh/OMS-2	MeOH	3.0	FFR	99.6	87	-	-	11
5	Pt/CeO_2-C	EtOH	2	FA	>99.9	77.1	7.3	11.7	12
6	Pt/CeO_2	H_2O	1.0	FFR	100	65.0	8.0	17.1	13
7	Pt/CeO_2	i-PrOH	3	FFR	> 99.9	53.6	3.6	39.0	14
8	$Cu-Mg_3AlO_{4.5}$	EtOH	6	FA	63.1	50.0	30.5	3.9	15
9	$Pt@Al_2O_3$	H_2O	$NaBH_4$	FA	>99	trace	75.6	24.4	16
				FFR	>99	trace	75.2	24.8	
10	Rh–Ir–ReOx/SiO_2	H_2O	6.0	FFR	>99.9	0.7	72.4	11.8	17
11	Pd–Ir–ReOx/SiO_2	H_2O	8	FFR	>99.9	1.4	71.4	4.4	18
12	Pt/Co_2AlO_4	EtOH	1.5	FFR	99.9	16.2	34.9	31.3	19
13	Ni(0)-La$(OH)_3$	i-PrOH	2	FFR	100	2.8	55.8	31.7	20
14	NiFeMgAl	EtOH	4	FA	99.7	25.9	31.0	23.2	21
15	Cu-Co–Al	EtOH	4	FA	98.8	16.1	41.6	15.0	22
16	$Cu-LaCO_3$	EtOH	6	FA	100	15.2	40.3	28.7	23
17	$Pt/MgAl_2O_4$	H_2O	4	FA	55.4	20.3	32.1	43.1	24
18	Ru–Mn/CNTs	H_2O	1.5	FA	81.8	16.5	-	43.7	25
19	CoAl-spinel	i-PrOH	4	FFR	-	1.8	40.3	42.1	26
20	$Cu-Al_2O_3$	EtOH	6	FA	85.8	48.1	22.2	2.7	27
21	CuMgAl	i-PrOH	6	FA	95.2	46	15.6	5.0	28
22	$Pt–Fe/MgTiO_3$	H_2O	1.0 / fixed-bed reactor	FFR	100	81	15	2	29
23	Pt/MgAl-LDHs@Al_2O_3	EtOH	3.0/ fixed-bed reactor	FA	96	86	6	6	30
24	Cu/MFI	EtOH	2.5/ fixed-	FA	99.5	16.0	69.2		31

			bed reactor						
25	Ni-Sn/ZnO	Isopropanol	4.0/fixed-bed reactor	FA	100	91	8.4	0.2	32
26	$LaNiO_3$	Isopropanol	3.0	FA	100		81	19	33

Table S4 Catalytic performances of various catalysts for the hydrogenolysis of Tetrahydrofuran-Dimethanol (THFDM)and HMF.

Entry	Catalysts	Solvent	H_2 Pressure (MPa)	Substrate	Conv. (%)	Sel. (%)			Ref.
						1,2,6-Hexanetriol	1,6-Hexanediol	1,2-Hexanediol	
1	$PtFe_{0.7}$/(Mg,Al)O	H_2O	1.0	HMF	100%	86.8	1.1	7.3	This work
2	10 wt% Pt-10 wt% WOx/TiO_2	H_2O	0.8	THFDM	6.4	>96	3	-	34
3	Pd/ZrP(7wt%Pd)	EtOH	formic acid	HMF	92.5	-	37.8	-	35
	Pd/ZrP(7wt%Pd)	EtOH	formic acid	HMF	96.9	-	42.5		

8. References

1 X. Ye, X. Y. Shi, J. Y. Li, B. B. Jin, J. Cheng, Z. H. Ren, H. Zhong, L. W. Chen, X. Liu, F. M. Jin, T. F. Wang, *Chem. Eng. J.* 2022, **440**,135844.

2 B. Zhang, H. Y. Yuan, Y. Liu, Z. J. Deng, M. Douthwaite, N. F. Dummer, R. J. Lewis, X. W. Liu, S. Luan, M. H. Dong, T. J. Wang, Q. L. Xu, Z. J. Zhao, H. Z. Liu, B. X. Han, G. J. Hutchings, *Nat. Commun.* 2024, ***15***: 7837.

3 B. Ravel, M. Newville, *J. Synchrotron Radiat* 2005, **12**, 537-541.

4 C. T. Wang, E. Guan, L. Wang, X. F. Chu, Z. Y. Wu, J. Zhang, Z. Y. Yang, Y. W. Jiang, L. Zhang, X. J. Meng, B. C. Gates, F. S. Xiao, *J. Am. Chem. Soc.* 2019, **141**, 8482-8488.

5 M. J. Frisch, G. W. Trucks, H. B. Schlegel, G. E. Scuseria, M. A. Robb, J. R. Cheeseman, G. Scalmani, V. Barone, G. A. Petersson, H. Nakatsuji, X. Li, M. Caricato, A. V. Marenich, J. Bloino, B. G. Janesko, R. Gomperts, B. Mennucci, H. P. Hratchian, J. V. Ortiz, A. F. Izmaylov, J. L. Sonnenberg, D. Williams-Young, F. Ding, F. Lipparini, F. Egidi, J. Goings, B. Peng, A. Petrone, T. Henderson, D. Ranasinghe, V. G. Zakrzewski, J. Gao, N. Rega, G. Zheng, W. Liang, M. Hada, M. Ehara, K. Toyota, R. Fukuda, J. Hasegawa, M. Ishida, T. Nakajima, Y. Honda, O. Kitao, H. Nakai, T. Vreven, K. Throssell, J. A. Montgomery, Jr., J. E. Peralta, F. Ogliaro, M. J. Bearpark, J. J. Heyd, E. N. Brothers, K. N. Kudin, V. N. Staroverov, T. A. Keith, R. Kobayashi, J. Normand, K. Raghavachari, A. P. Rendell, J. C. Burant, S. S. Iyengar, J. Tomasi, M. Cossi, J. M. Millam, M. Klene, C. Adamo, R. Cammi, J. W. Ochterski, R. L. Martin, K. Morokuma, O. Farkas, J. B. Foresman, and D. J. Fox, *Gaussian 16, Revision A.03*, Gaussian, Inc., Wallingford CT, 2016.

6 A. V. Marenich, C. J. Cramer, D. G. Truhlar, *J. Phys. Chem. B.* 2009, **113**, 6378-6396.

7 S. Grimme, *J. Comput. Chem.* 2006, **27**, 1787-1799..

8 F. Neese, F. Wennmohs, U. Becker, C. Riplinger, *J. Chem. Phys.* 2020, **152**.

9 T. Mizugaki, T. Yamakawa, Y. Nagatsu, Z. Maeno, T. Mitsudome, K. Jitsukawa, K. Kaneda, *ACS. Sustain. Chem. Eng.* 2014, **2**, 2243-2247.

10 P. P. Upare, Y. Kim, K. R. Oh, S. J. Han, S. K. Kim, D. Y. Hong, M. Lee, P. Manjunathan, D. W. Hwang, Y. K. Hwang, *ACS. Sustain. Chem. Eng.* 2021, **9**, 17242-17253.

11 Pisal, D. S.; Yadav, G. D. Single-Step Hydrogenolysis of Furfural to 1,2-Pentanediol Using a Bifunctional Rh/OMS-2 Catalyst. *ACS. Omega* 2019, **4**, 1201-1214.

12 T. Tong, X. H. Liu, Y. Guo, M. N. Banis, Y. F. Hu, Y. Q. Wang, *J. Catal.* 2018, **365**, 420-428.

13 R. F. Ma, X. P. Wu, T. Tong, Z. J. Shao, Y. Q. Wang, X. H. Liu, Q. N. Xia, X. Q. Gong, *ACS. Catal.* 2017, **7**, 333-337.

14 T. Tong, Q. N. Xia, X. H. Liu, Y. Q. Wang, *Catal. Commun.* 2017, **101**, 129-133.

15 H. L. Liu, Z. W. Huang, F. Zhao, F. Cui, X. M. Li, C. G. Xia, J. Chen, *Catal. Sci. Technol.* 2016, **6**, 668-671.

16 J. Y. Yeh, B. M. Matsagar, S. S. Chen, H. L. Sung, D. C. W. Tsang, Y. P. Li, K. C. W. Wu, *J. Catal.* 2020, **390**, 46-56.

17 S. B. Liu, Y. Amada, M. Tamura, Y. Nakagawa, K. Tomishige, *Catal. Sci. Technol.* 2014, **4**, 2535-2549.

18 S. B. Liu, Y. Amada, M. Tamura, Y. Nakagawa, K. Tomishige, *Green Chem.* 2014, **16**, 617-626.

19 W. J. Xu, H. F. Wang, X. H. Liu, J. W. Ren, Y. Q. Wang, G. Z. Lu, *Chem. Commun.* 2011, **47**, 3924-3926.

20 H. W. Wijaya, T. Sato, H. Tange, T. Hara, N. Ichikuni, S. Shimazu, *Chem. Lett.* 2017, **46**, 744-746.

21 Y. W. Shao, J. Z. Wang, K. Sun, G. M. Gao, C. Li, L. J. Zhang, S. Zhang, L. L. Xu, G. Z. Hu, X. Hu, *Renew Energ.* 2021, **170**, 1114-1128.

22 T. P. Sulmonetti, B. Hu, S. Lee, P. K. Agrawal, C. W. Jones, *ACS. Sustain. Chem. Eng.* 2017, **5**, 8959-8969.

23 F. F. Gao, H. L. Liu, X. Hu, J. Chen, Z. W. Huang, C. G. Xia, *Chinese J. Catal.* 2018, **39**, 1711-1723.

24 X. S. Li, J. F. Pang, J. C. Zhang, X. Q. Li, Y. Jiang, Y. Su, W. Z. Li, M. Y. Zheng, *Catalysts* 2021, **11**, 415.

25 X. L. Wang, Y. J. Weng, X. L. Zhao, X. X. Xue, S. H. Meng, Z. F. Wang, W. B. Zhang, P. G. Duan, Q. Sun, Y. L. Zhang, *Ind. Eng. Chem. Res.* 2020, **59**, 17210-17217.

26 L. Gavila, A. Lahde, J. Jokiniemi, M. Constanti, F. Medina, E. del Rio, D. Tichit, M. G. Alvarez, *Chemcatchem* 2019, **11**, 4944-4953.

27 H. L. Liu, Z. W. Huang, H. X. Kang, C. G. Xia, J. Chen, *Chinese J. Catal.* 2016, **37**, 700-710.

28 Y. W. Shao, J. Z. Wang, H. N. Du, K. Sun, Z. M. Zhang, L. J. Zhang, Q. Y. Li, S. Zhang, Q. Liu, X. Hu, *ACS. Sustain. Chem. Eng.* 2020, **8**, 5217-5228.

29 C. Cao, W. X. Guan, Q. Y. Liu, L. Li, Y. Su, F. Liu, A. Q. Wang, T. Zhang, *Green Chem.* 2024, **26**, 6511-6519.

30 Y. R. Zhu, W. F. Zhao, J. Zhang, Z. An, X. D. Ma, Z. J. Zhang, Y. T. Jiang, L. R. Zheng, X. Shu, H. Y. Song, X. Xiang, J. He, *ACS. Catal.* 2020, **10**, 8032-8041.

31 D. F. Dai, C. Feng, M. M. Wang, Q. Z. Du, D. D. Liu, Y. Pan, Y. Q. Liu, *Catal. Sci. Technol.* **2022**, *12*, 5879-5890.

32 A. S. Nimbalkar, K. R. Oh, D. Y. Hong, B. G. Park, M. Lee, D. W. Hwang, A. Awad, P. P. Upare, S. J. Han, Y. K. Hwang, *Green. Chem.* 2024. DOI: 10.1039/D4GC02757D.

33 Y. S. Zhang, H. Y. Xue, M. Q. Cheng, X. M. Yang, Z. Zhang, X. R. Zhao, A. Rezayan, D. D. Han, D. Wu, C. B. Xu, *ACS. Catal.* 2024, **14**, 10009-10021.

34 J. Y. He, S. P. Burt, M. Ball, D. T. Zhao, I. Hermans, J. A. Dumesic, G. W. Huber, *ACS. Catal.* 2018, **8**, 1427-1439.

35 J. Tuteja, H. Choudhary, S. Nishimura, K. Ebitani, *Chemsuschem* 2014, **7**, 96-100.
